# Transcriptomics in the tropics: Total RNA-based profiling of Costa Rican bromeliad-associated communities

**DOI:** 10.1016/j.csbj.2014.12.001

**Published:** 2014-12-13

**Authors:** Shana K. Goffredi, Gene E. Jang, Mohamed F. Haroon

**Affiliations:** a Occidental College, United States; b University of Queensland, Australia

**Keywords:** Bromeliad, Metatranscriptome, Methanogen, Rainforest, Diversity

## Abstract

RNA-Seq was used to examine the microbial, eukaryotic, and viral communities in water catchments (‘tanks’) formed by tropical bromeliads from Costa Rica. In total, transcripts with taxonomic affiliation to a wide array of bacteria, archaea, and eukaryotes, were observed, as well as RNA-viruses that appeared related to the specific presence of eukaryotes. Bacteria from 25 phyla appeared to comprise the majority of transcripts in one tank (Wg24), compared to only 14 phyla in the other (Wg25). Conversely, eukaryotes from only 16 classes comprised the majority of transcripts in Wg24, compared to 24 classes in the Wg25, revealing a greater eukaryote diversity in the latter. Given that these bromeliads had tanks of similar size (i.e. vertical oxygen gradient), and were neighboring with presumed similar light regime and acquisition of leaf litter through-fall, it is possible that pH was the factor governing these differences in bacterial and eukaryotic communities (Wg24 had a tank pH of 3.6 and Wg25 had a tank pH of 6.2). Archaeal diversity was similar in both tanks, represented by 7 orders, with the exception of Methanocellales transcripts uniquely recovered from Wg25. Based on measures of FPKG (fragments mapped per kilobase of gene length), genes involved in methanogenesis, in addition to a spirochaete flagellin gene, were among those most highly expressed in Wg25. Conversely, aldehyde dehydrogenase and monosaccharide-binding protein were among genes most highly expressed in Wg24. The ability to observe specific presence of insect, plant, and fungi-associated RNA-viruses was unexpected. As with other techniques, there are inherent biases in the use of RNA-Seq, however, these data suggest the possibility of understanding the entire community, including ecological interactions, via simultaneous analysis of microbial, eukaryotic, and viral transcripts.

## 1 Introduction

In the Neotropics, plants within the family Bromeliaceae are important epiphytes due to their relative abundance and to their compact foliar-based catchments (or ‘tanks’) capable of retaining water. Because epiphytic bromeliads virtually lack absorptive roots, these tanks provide the plants with water and nutrients released by heterotrophic decomposition of impounded debris [2], [46]. Tank bromeliads can contain as much as 50,000 l of suspended water ha^− 1^ of canopy [8], a habitat that has potential significance with regard to local biodiversity and community structure. It has been shown that increased environmental complexity results in increased species diversity for both bacteria and animals [21], [43]. This may be particularly true for bromeliads, which not only contribute to the heterogeneity of the forest, but themselves are complex structures with compartments of different sizes and unique physical–chemical characteristics [1], [24]. Faunal abundance and diversity are enhanced in bromeliad tanks, which house up to 100 × greater animal densities than surrounding forest floor habitats [34], [39], [40]. A vast array of aquatic taxa take advantage of these catchments, including numerous insect families, ostracods, copepods, rotifers, and amphibians, as well as protozoans, algae, fungi, bacteria, and archaea [3], [9], [11], [12], [14], [28], [38].

Due to their small size, defined boundaries, and relatively simple faunal communities, bromeliad catchments are ideal for the investigation of whole ecosystem complexity, a notoriously difficult task for lakes and other large, more complex freshwater habitats [24]. As a consequence, bromeliad habitats have been used for the study of carbon and nitrogen cycling, food web dynamics, and even the effects of warming on trophic cascades [20], [27], [30], [32], [33]. Similarly, bromeliad tanks have been investigated for their unique bacterial and archaeal communities, as well as the influence of various ‘micro-limnological’ parameters, such as pH, oxygen, dissolved organic carbon, and light, on the microbial communities [11], [12], [15], [30]. pH conditions, for example, can have a major impact on the structure of freshwater microbes [17], and in a study of Costa Rican bromeliads it was discovered that Alphaproteobacteria, Acidobacteria, Planctomycetes, and Bacteroidetes dominated tanks of low pH (< 5.1), while Betaproteobacteria, Firmicutes, and Bacteroidetes dominated tanks of high pH (> 5.3), based on 16S rRNA gene surveys [11]. The influence of pH was not observed for archaea [12] and is generally less resolved for animal communities [4], [7]. Petrin and colleagues determined that the ability of stream invertebrates to adapt to naturally low pH conditions was variable among different taxonomic groups and that total species richness and abundance did not change with pH [35].

With a new appreciation for the intertwined worlds of metazoans and microbes, it is an exciting prospect to explore the possibility of examining the entire community (from viruses to eukaryotes) with a single technique. The current state of knowledge with regard to plant-associated aquatic habitats is based on the counting of animals or protozoa or, in the case of bacteria and archaea, based on 16S rRNA ribosomal surveys (ex. [11], [12], [14], [28], [38], [42]). There have been no papers published on viral communities within these abundant and important ecosystems. Each of these studies, like most, was conducted with a specific organismal domain in mind. Despite decades of investigation, total community dynamics has not been measured for any given bromeliad tank community. Additionally, DNA-based analysis (ex. 16S rRNA gene surveys), although a technique that has yielded much new information about the living world, does little to inform our understanding of functional microbial status and activity in situ. Metatranscriptomic analysis (the study of the RNA from the entire community of organisms), however, allows for detection of mRNA transcripts from all domains, rather than specific taxa. Advances in high-throughput sequencing and bulk extraction of mRNA allow for RNA-based studies of organismal functioning in complex environments. Despite certain technological challenges, microbial metatranscriptomics has already helped elucidate microbial responses to oil spills and the resulting deep-sea hydrocarbon plumes, differences between anoxic and oxic paddy soils, and metabolic activity of methane-producing microbes, to name a few [31], [41], [47]. Similarly, metatranscriptomic approaches have been used to investigate the functional diversity of the eukaryotic microorganisms within the rumen of muskoxen [37] and in forest soils [6], [44]. Notably, Urich et al. obtained information on the structure and function of organisms from all three domains of life, in a single RNA-centered metatranscriptomic experiment with forest soil [44]. While no single ‘omics’ approach is able to elucidate the truly complex nature of entire communities, the current results suggest the possibility of using similar RNA-based analyses to profile total community dynamics, including eukaryotes, archaea, bacteria, and possibly viruses. We provide here preliminary data on the diversity of total communities associated with two bromeliad tanks from La Selva Biological Station in a lowland tropical rainforest in Costa Rica.

## 2 Methods

### 2.1 Sample collection

Bromeliad tank debris and water were collected from two *Werauhia gladioliflora* specimens in La Selva Biological station, a wet (4 m annual rainfall) lowland neotropical reserve located in northeastern Costa Rica (10°25′52″ N, 84°00′12″ W). Samples were collected in June 2012, from two plants, neither of which was in bloom, growing at heights of ~ 2.0 m from the ground. The bromeliads had tanks of ~ 10 cm depth (i.e. similar vertical oxygen gradients), and were neighboring with presumed similar light regime and acquisition of leaf litter through-fall. Since previous studies revealed strong correlations between bacterial diversity and bromeliad tank pH, which can range from pH 3 to 7 [11], two tanks at the pH extremes were chosen for comparison of gene expression from all 3 domains of life, in addition to viruses. At the time of sample collection, ‘Wg24’ had a tank pH of 3.56, whereas ‘Wg25’ had a pH of 6.21. The entire tank contents were collected from the deepest horizons within the central axil of each plant, and put into clean, RNase-free 15 ml conical tubes for transport to the lab. Tank samples were centrifuged at 15,000 rpm for 10 min, water was removed, and the remaining debris was resuspended in 3 × LifeGuard Soil Preservation Solution (MoBio Laboratories), and stored at − 80 °C for further analysis. Samples were at room temperature in the LifeGuard solution for ~ 15 h during air transport back to Occidental College.

### 2.2 Total nucleic acid extraction

Nucleic acid extraction was performed at Occidental College, using RNase-free materials (tubes and pipette tips) on workstations treated with RNase*Zap* Wipes (Ambion). The RNA PowerSoil Total RNA Isolation Kit (MoBio Laboratories) was used for bulk extraction of RNA, with slight protocol modifications to extract sufficient quantities of nucleic acid for metatranscriptomic analysis (~ 90–120 ng μl^− 1^). Modifications included the use of freshly prepared low-pH phenol:chloroform:isoamyl alcohol (25:24:1 ratio, respectively) for nucleic acid extraction (PowerSoil Step 5), a room temperature incubation for secondary debris precipitation (PowerSoil Step 9), gentle encouragement of fluid flow through the RNA capture column using a syringe (PowerSoil Steps 16–19), and an overnight nucleic acid precipitation step (PowerSoil Step 20). Successful nucleic acid extraction was verified by NanoDrop UV spectrophotometry, ensuring sufficient RNA concentrations and purity. RNA extraction was immediately followed by removal of contaminant DNA from the extracted RNA, using TURBO DNA-free DNase Treatment and Removal Reagents (Ambion). A treatment of 4 Units TURBO DNase per 100 μl RNA was applied, with a 20 min 37 °C incubation for sufficient DNA contaminant removal.

### 2.3 RNA preparation and sequencing

Total extracted RNA was submitted to Otogenetics Corporation (Norcross, GA USA) for RNA-Seq analysis. Briefly, 9–12 μg of total RNA was subjected to rRNA depletion using the RiboZero Meta-Bacteria kit (Epicentre Biotechnologies) and cDNA was generated from the depleted RNA using the NEBNext mRNA Sample Prep kit (New England Biolabs). Illumina libraries were created from the cDNA using NEBNext reagents (New England Biolabs). The quality, quantity and size distribution of the Illumina library products were determined using an Agilent Bioanalyzer 2100. The libraries were then submitted for Illumina HiSeq2000 sequencing according to standard operations.

### 2.4 Bioinformatic analysis

Paired-end 100 nucleotide (nt) reads were generated and checked for data quality using FASTQC (Babraham Institute). For removal of ribosomal RNA sequences (5S, 16S, 23S and the eukaryote rRNA equivalents) that were not subtracted via RiboZero depletion, SortMeRNA software [25] was used to filter by comparison against the Silva rRNA archaea, bacteria and eukaryotic rRNA databases [36]. For Wg24, 13,562,864 remained after rRNA removal (78%), out of 17,258,552 initial reads. For Wg25, 10,761,162 remained after rRNA removal (67%), out of 15,998,012 initial reads. These ~ 10–14 million 100 nt reads were assembled into larger contigs (500–5000 nt in length) using CLC Genomics Workbench v.6.0.1 (2533 and 1938 contigs were generated for Wg24 and Wg25, respectively). Sequencing reads were quality filtered and Illumina adapters were trimmed using the CLC Genomics Workbench with a quality threshold of 0.01. Trimmed reads were assembled de novo using the CLC Genomics Workbench using an automatic kmer size of 22 and a bubble size of 50. To assign function to the assembled metatranscriptomic contigs, Prodigal v.2.60 was used to call open reading frames (ORFs; [22]), which were annotated functionally and taxonomically by blastp, using an E-value of < 10^− 5^ against the Integrated Microbial Genomes (IMG) system v.4.0 [29] which includes archaeal, bacterial and eukaryotic full and draft genomes, as well as viral and environmental sourced genes. Of the 2533 contigs within library Wg24, 434 had functional gene annotations without a taxonomic affiliation, while an additional 166 had no homologs in public databases. Similarly, of the 1938 contigs within library Wg25, 128 had functional gene annotations without a taxonomic affiliation, while an additional 88 had no homologs in public databases. For quantification of gene expression, rRNA-subtracted mRNA sequences were mapped against the assembled metatranscriptome using BWA v.0.6.2 [26]. The number of reads that mapped against a particular gene was normalized by the length of the gene in order to generate an FPKG value (number of fragments mapped per kilobase of gene length), using the sam2fpkg.pl script v.0.3 (http://github.com/minillinim/sam2fpkg; [16]). To allow analysis of differential gene expression between samples, the FPKG values were then normalized by the total read length and multiplied by a million, for ease of comparison. The transcript reads were deposited in the NCBI Sequence Read Archive under accession numbers SRR1697355 (Wg24) and SRR1697356 (Wg25).

## 3 Results and discussion

Metatranscriptomic analysis (the study of RNA from the entire community of organisms) was performed on the water catchments formed by tropical bromeliads from a Costa Rican rainforest. The community associated with Costa Rican tropical bromeliads was deduced through affiliation of non-ribosomal transcript sequences with particular taxa in public databases. Overall results from two bromeliad tanks indicate the presence of genes from 25 bacterial phyla (including 4 classes within the Proteobacteria), 7 archaeal orders, 21 metazoan classes (including 6 orders of insect), 8 fungal classes, as well as viruses and transcripts related to those recovered from environments, including plant biomass reactors, freshwater lakes, and the rhizosphere (Table 1, Table 2, Table 3). There was a notable difference in the diversity of each taxonomic domain between the two tanks. For example, bacteria from 25 phyla appeared to comprise the majority of transcripts in Wg24, compared to only 14 phyla in Wg25 (Table 1, Table 2). Even after accounting for a difference in total reads analyzed (17.2 versus 16.0 million reads, respectively) or contigs recovered (2531 versus 1938, respectively), this represented a greater bacterial diversity within tank Wg24 (Fig. S1). Conversely, eukaryotes from only 16 classes comprised the majority of transcripts in Wg24, compared to 24 eukaryote classes in Wg25 (Table 3), revealing a greater overall eukaryote diversity at less acidic conditions. Archaeal diversity was similar in both tanks, represented by 7 orders, with the exception of Methanocellales transcripts uniquely recovered from Wg25 (Table 1, Table 2). Additionally, some transcripts were annotated as environmentally-derived, from uncultured microbes inhabiting aquatic sediments (e.g. Sakinaw Lake, Lake Washington), bioreactors (e.g. *Poplar* biomass reactors, enrichment cultures), and associated with eukaryotes (e.g. Costa Rican termite hindgut microbiome, *Arabidopsis* rhizosphere communities; Table 1). Viral transcripts were recovered from both tanks and included insect, plant, and fungi-associated RNA-viruses. Specific results from each of these organismal levels are discussed below.

Bromeliad eukaryote communities are abundant and diverse, typically dominated by annelids and larvae of aquatic insects, including many species of Coleoptera and Diptera [23], [24], [28], [38]. Our RNA-based results are consistent with these previous studies and suggest that certain biota are possibly adapted to more or less acidic conditions in these unique, plant-based aquatic habitats. For example, the higher pH Wg25 tank was host to a large and varied number of eukaryotic RNA transcripts, including many closely related to those of annelids (24% of the total eukaryote transcripts) and insects (37% of total eukaryote transcripts; e.g., Diptera, Hymenoptera, Coleoptera, Hemiptera, Phthiraptera, and Lepidoptera). Additional eukaryote transcripts from Wg25 included members of the Branchiopoda, Cnidaria, Mollusca, Nematoda, and Placozoa, to name a few (Table 2). Whether these transcripts reflect the actual presence of similar organisms, or whether they are affiliated to these groups, in silico, as limitations of existing databases, remains to be seen. Additionally, chordate-sourced transcripts were present in Wg25 and likely reflected the common transient use of bromeliad tanks for food, water and shelter by monkeys, birds, and amphibians ([3]; SKG, pers. obs). Interestingly, transcripts affiliated with a chytrid fungus were only recovered from Wg25, from where we also recovered amphibian-affiliated transcripts, most likely the blue-jeans frog *Oophaga pumilio*, which commonly uses bromeliad tanks as nurseries [45]. On the other hand, eukaryote transcripts were lower in diversity within Wg24, possibly reflecting the putatively more stressful conditions of the acidic catchment (Table 3). Far fewer eukaryote groups were represented and of the insect-sourced transcripts in Wg24 sample, 61% were associated with Coleoptera, while annelid transcripts were comparatively absent (Table 1). Fungal transcripts made up the remaining minority in Wg24, including those from 4 classes (Table 3), likely involved in the hydrolysis of plant material trapped in the tanks. Many eukaryotic viral transcripts were detected in both bromeliad tanks, and mirrored the diverse eukaryotic community, including many likely derived from plant, insect, and fungal (yeast, water mold) dsRNA and ssRNA viruses.

Transcripts from numerous bacterial phyla (including 4 classes within the Proteobacteria), were recovered from the bromeliad tanks, comprising 25 phyla in Wg24 and a subset of these (~ 14 phyla) from Wg25 (Table 1). Both tanks contained numerous transcripts belonging to the Firmicutes, in relatively similar numbers (13–18% of total bacterial transcripts recovered), but much higher diversity from Wg24 (102 species represented versus 48 species, respectively; Table 2). The greater diversity in Wg24 was generally consistent among all bacterial groups (Table 2; Fig. S1), even after normalization of sequencing effort and contig yield, which was initially ~ 30% higher in Wg24. Notable differences included the dominance of Acidobacteria in Wg24 (which comprised 26% of the total bacterial transcripts recovered), versus the dominance of Gammaproteobacteria in Wg25 (60% of the total bacterial transcripts recovered). Additional transcripts recovered mainly from Wg24 included those affiliated with Spirochaetes and Alphaproteobacteria, the latter of which were exceptionally diverse in Wg24, compared to Wg25 (transcripts from 48 species compared to 9 species, respectively; Table 2). Both of these bacterial groups are well known to tolerate more acidic conditions (consistent with Wg24), and a previous 16S rRNA-based study on Costa Rican bromeliad tanks demonstrated pH-dependent structure within the bacterial community, including the dominance of Acidobacteria and Alphaproteobacteria in bromeliad tanks of pH < 5.0 [12]. Additionally, consistent with different pH regimes, transcripts annotated as ‘Environmental’ from the low pH tank Wg24 resembled those recovered from a Poplar biomass reactor, while the transcripts from the higher pH Wg25 tank resembled those recovered from typical freshwater lakes (Lake Sakinaw and Lake Washington; [10]). The diverse nature of these related habitats is consistent with the duality of the highly stratified bromeliad tank environment, which has many lake-like qualities in the upper less acidic layers, and the decompositional anoxic nature in the deeper, more acidic layers. Although not well constrained, factors that affect the distribution and abundance of the bromeliads themselves, such as rainfall, light, and temperature, also likely have both direct and indirect effects on communities within the tanks [13].

The metatranscriptome data for archaeal presence and diversity also followed the same pattern as recovered from previous DNA-based studies. Transcripts with archaeal taxonomic assignments were present at similar levels in both bromeliad tanks, comprising ~ 7% of the transcript reads. Archaeal diversity was similar in both tanks, represented by 7 orders, with the exception of Methanocellales transcripts uniquely recovered from Wg25 (Table 1, Table 2). Both of the archaeal populations were dominated by the order Methanomicrobiales (86–95% of the recovered archaeal transcripts; Table 1), involved in hydrogenotrophic methanogenesis. Previous 16S rRNA gene analysis of Costa Rican bromeliad catchments also revealed that archaea were dominated (up to 90% of 16S rRNA genes) by the hydrogenotrophic methanogens (Methanomicrobiales and Methanocellales; [11]).

## 4 Functional nature of the community

Bacterial communities play essential roles in energy and matter flow in aquatic microcosms, through detrital mineralization and nutrient release. Of particular interest is the microbial role in nitrogen and carbon-cycling in bromeliad tanks, due to the abundance of carbon-based leaf litter, animal carcasses, and feces that constitute the organic detritus. Despite their status as one of the most abundant aquatic ecosystems in the world, and their classification as true microcosms (with high variability among physical, biological, and chemical parameters), remarkably little is known about the function of bromeliad-associated microbial communities [15], [18]. Genes relevant to carbon turnover, including chitinase and methyl coenzyme M reductase (involved in archaeal methanogenesis) have been recovered from bromeliad communities [11]. Claims have even been made that bromeliad tanks also serve as significant sites for global methane production by archaeal microbes [30]. In the current study, measures of fragments mapped per kilobase of gene length (FPKG) revealed genes involved in methanogenesis to be among those most highly expressed only in Wg25 (Table 4), suggesting the differential presence versus activity of methanogens in this unique habitat. A dehydrogenase associated with Acidobacteria, and numerous Spirochaete-related genes ranked among the most expressed in Wg24 (Table 4). The challenge posed by acidic environs might necessitate cellular defense mechanisms in microorganisms that inhabit such niches [5]. Increased expression of genes involved in proton pumping, repair of degraded macromolecules, and transcriptional and metabolic alterations was observed in the Wg24 library (data not shown). For example, gene transcripts for superoxide dismutase and peroxidase, enzymes necessary for the neutralization of toxic superoxide (O_2_^−^) and hydrogen peroxide metabolites, and small heat shock proteins, were almost exclusively detected in Wg24. Together, such differences in genes relevant to cellular defense and response mechanisms perhaps reflect the high-stress, low-pH environment in this particular bromeliad catchment.

## 5 Summary and outlook

Freshwater tropical ecosystems, including bromeliad tanks, support disproportionate levels of biodiversity, especially microorganisms and invertebrates, relative to their spatial coverage, and are expected to be highly vulnerable to habitat perturbations resulting from climate change [19]. Here we present the results of a RNA-centered metatranscriptomic comparison of tank-associated community structure and function between two neighboring bromeliads. While no single ‘omics’ approach is able to elucidate the truly complex nature of entire communities, these results suggest the possibility of using similar RNA-based analyses to profile total community dynamics, including eukaryotes, archaea, bacteria, and RNA viruses. The comparison of bromeliad tanks of similar size, but different pH conditions, revealed different communities encompassing all domains of life, as well as viruses. Interestingly, the diversity of animal mRNA transcripts was lower in the low pH tank, with insects primarily dominating. Despite other potential variables shaping these communities, it is possible that the specific and permanent reduction in pH that is expected to accompany future increases in CO_2_ on the planet could result in the loss of biodiversity within bromeliad tanks over time. In order to confirm this hypothesis, it will be necessary to conduct a more thorough analysis of the structure and function of organisms in these habitats.

The following is the supplementary data related to this article.

Supplemental Fig. 1 Number of bacterial species represented in metatranscriptomic libraries from two bromeliad tanks. For Wg25, with a pH of 6.2, species number for each phylum was multiplied by 1.3 (from Table 2) to account for a difference in total reads analyzed (16.0 million versus 17.2 million reads, respectively) or contigs recovered (1938 versus 2531), as compared to Wg24 with a pH of 3.6, respectively.

## Figures and Tables

**Table 1:** Distribution of assignments of all RNA transcripts attributed to bacteria, archaea, eukaryote, environmental or viruses (listed by percent of the category).

Closest taxonomic match	% of category^a^		Closest taxonomic match	% of category^a^	
Wg24	Wg25	Wg24	Wg25
**Bacteria**	**1539**	**858**	**Eukarya**	**215**	**736**
Acidobacteria	26	1	Insecta	82	37
Firmicutes	13	18	Chordata	2	12
Gammaproteobacteria	10	60	Fungi^b^	4	1
Spirochaetes	10	1	Annelida	< 1	24
Alphaproteobacteria	10	1	Crustacea	0	12
Bacteroidetes	7	5	Arachnida	0	3
Verrucomicrobia	4	4	Other^c^	13	20
Deltaproteobacteria	4	2	**Environmental**	**539**	**171**
Chloroflexi	3	2	Poplar biomass reactor	26	13
Betaproteobacteria	3	2	Sakinaw Lake	17	34
Actinobacteria	2	< 1	Lake Washington	13	6
Cyanobacteria	2	< 1	Arabidopsis	9	3
Planctomycetes	1	0	Termite hindgut	7	2
Other^d^	15	5	Volcanic mats	7	11
**Viruses**	**37**	**36**	Fiber fraction	7	9
Insect host	57	67	Other^e^	14	21
Plant host	22	17	**Archaea**	**177**	**126**
Fungal host	8	3	Methanomicrobiales	95	86
Other host^f^	13	13	Methanocellales	0	11
		Other^g^	5	3

a Values in bold represent the total number of RNA transcripts for each category, out of a total of 2531 (Wg24 = pH 3.6) and 1938 (Wg25 = pH 6.2).

b Inlcudes Ascomycota, Basidiomycota, Blastocladiomycota, and Chytridiomycota.

c Represents other eukaryote classes, including Branchiopoda, Cnidaria, Mollusca, Nematoda, Placozoa, and Porifera.

d Represents other bacterial phyla, including Aquificae, Chlorobi, Deferribacteres, and Ignavibacteria.

e Includes environments such as the wild Panda gut, marine sediments, and other biomass reactors.

f Includes protist, crustacean, avian and mammalian viruses.

g Represents other archaeal orders, including Methanosarcinales, Thermoproteales, Halobacteriales, Thermococcales, and Desulforococcales.

**Table 2:** Diversity distribution of assignments of all RNA transcripts attributed to bacteria and archaea, at the level of order, family, genus, and species.

Closest taxonomic match	No. representative groups, from transcripts (order–family–genus–species)^a^							
Wg24				Wg25			
Order	Family	Genus	Species	Order	Family	Genus	Species
**Bacteria**^b^	**90**	**170**	**333**	**450**	**33**	**71**	**112**	**169**
Acidobacteria	4	5	7	8	4	4	4	4
Firmicutes	6	26	66	102	8	16	23	48
Gammaproteobacteria	13	19	44	66	4	5	16	38
Spirochaetes	2	5	9	17	2	2	4	7
Alphaproteobacteria	4	16	41	48	4	6	9	9
Bacteroidetes	4	14	38	46	4	9	23	24
Verrucomicrobia^c^	4	5	4	5	4	4	1	4
Deltaproteobacteria	6	15	21	30	5	8	10	13
Chloroflexi	8	9	10	12	4	4	4	4
Betaproteobacteria	6	10	25	31	2	4	9	11
Actinobacteria	4	17	21	27	2	2	2	2
Cyanobacteria	6	13	15	19	1	1	1	1
Planctomycetes	4	5	10	10	0	0	0	0
Other^d^	18	18	31	35	7	7	8	8
**Archaea**	**4**	**6**	**13**	**14**	**3**	**7**	**9**	**14**
Methanomicrobiales^e^	–	4	5	6	–	4	5	6
Methanocellales	–	0	0	0	–	3	1	1
Other^f^	5	7	8	8	3	3	3	5

– = at the order level already.

a Total number of RNA transcripts was 2531 (Wg24 = pH 3.6) and 1938 (Wg25 = pH 6.2).

b Listed in order of abundance, in the low pH tank, from Table 1.

c Most Verrucomicrobia genera are unclassified.

d Represents 15 other phyla, including Aquificae, Chlorobi, Deferribacteres, and Ignavibacteria.

e Most Methanomicrobia genera are unclassified.

f Represents other archaeal orders, including Methanosarcinales, Thermoproteales, Halobacteriales, Thermococcales, and Desulforococcales.

**Table 3:** Differential presence of functional transcripts from various animal and fungal groups.

Group^a^	Wg24	Wg25
Insecta	Coleoptera	Coleoptera
Diptera	Diptera
Hemiptera	Hemiptera
Hymenoptera	Hymenoptera
Lepidoptera	Lepidoptera
Phthiraptera	Phthiraptera
Arachnida	–	Ixodida
Branchiopoda	–	Diplostraca
Annelida	Hirudinea	Hirudinea
–	Polychaeta
Chordata	–	Actinopterygii
–	Amphibia
–	Appendicularia
Ascidiacea	Ascidiacea
–	Branchiostoma
Mammalia	Mammalia
Cnidaria	Anthozoa	Anthozoa
Hydrozoa	Hydrozoa
Mollusca	–	Gastropoda
Nematoda	Chromadorea	Chromadorea
Placozoa	–	Unclassified
Porifera	Demospongiae	Demospongiae
Ascomycota	–	Dothideomycetes
Eurotiomycetes	Eurotiomycetes
–	Saccharomycetes
–	Schizosaccharomyces
Sordariomycetes	Sordariomycetes
Basidiomycota	Agaricomycetes	Agaricomycetes
Exobasidiomycetes	–
Blastocladiomycota	Blastocladiomycetes	–
Chytridiomycota	–	Chytridiomycetes

– = not present.

a All animal phyla are designated by class level. Fungal groups are shown by orders.

**Table 4:** Top 10 most highly expressed bacterial and archaeal functional transcripts based on fragments mapped per kilobase of gene length (FPKG), as annotated by IMG, and not including hypothetical genes.

Functional gene	Organism	FPKG	FPKG normal^a^
*Wg24*			
Aldehyde dehydrogenase (NAD)	*Acidobacterium capsulatum*	25,799	1902
Coenzyme-B sulfoethylthiotransferase (i.e. methyl-coenzyme M reductase)	*Methanoregula boonei*	18,004	1327
Monosaccharide-binding protein	*Spirochaeta aurantia*	10,866	801
Copper amine oxidase-like protein	*Clostridium thermocellum*	10,117	746
5,10-Methylenetetrahydromethanopterin reductase	*Methanoregula formicicum*	9453	697
Chaperonin GroL	*Thermaerobacter subterraneus*	8523	628
Cytoplasmic filament protein A	*Spirochaeta smaragdinae*	7818	576
Periplasmic sugar-binding protein	*Spirochaeta caldaria*	7211	532
Flagellin domain protein	*Spirochaeta smaragdinae*	4296	317
Coenzyme F420 hydrogenase	*Methanoregula boonei*	3864	285

*Wg25*			
Periplasmic flagellin	*Leptospira interrogans*	2,541,718	236,194
Methyl-coenzyme M reductase operon D	*Methanoregula formicicum*	8868	824
Methyl-coenzyme M reductase operon C	*Methanoregula formicicum*	7705	716
Methyl-coenzyme M reductase, g subunit	*Methanoregula boonei*	6525	606
Elongation factor Ts	*Flavobacteria* sp. BAL38	6373	592
Methyl-coenzyme M reductase, b subunit	*Methanoregula formicicum*	5432	505
Glyceraldehyde-3-phosphate dehydrogenase	*Enterobacter cancerogenus*	5355	498
Methyl-coenzyme M reductase, a subunit	*Methanolinea tarda*	4799	446
Cytochrome c oxidase, subunit III	*Rhodopseudomonas palustris*	3869	360
Coenzyme F420 hydrogenase	*Methanoregula boonei*	3780	351

a FPKG values were normalized within each library (raw FPKG/rRNA subtracted reads ∗ 1,000,000) in order to compare between libraries.

## References

[1] Araújo V.A., Melo S.K., Araújo A.P.A., Gomes M.L.M., Carneiro M.A.A. (2007). Relationship between invertebrate fauna and bromeliad size Brazilian. J Biol.

[2] Benzing D.H. (1970). Foliar permeability and the absorption of minerals and organic nitrogen by certain tank bromeliads. Bot Gaz.

[3] Benzing D.H. (2000). Bromeliaceae: profile of an adaptive radiation.

[4] Collier K.J., Ball O.J., Graesser A.K., Main M.R., Winterbourn M.J. (1990). Do organic and anthropogenic acidity have similar effects on aquatic fauna?. Oikos.

[5] Cotter P.D., Hill C. (2003). Surviving the acid test: responses of gram-positive bacteria to low pH. Microbiol Mol Biol Rev.

[6] Damon C., Lehembre F., Oger-Desfeux C., Luis P., Ranger J. (2012). Metatranscriptomics reveals the diversity of genes expressed by eukaryotes in forest soils. PLoS One.

[7] Dangles O., Malmqvist B., Laudon H. (2004). Naturally acid freshwater ecosystems are diverse and functional: evidence from boreal streams. Oikos.

[8] Fish D., Frank J.H., Lounibos L.P. (1983). Phytotelmata: terrestrial plants as hosts for aquatic insect communities. Phytotelmata: flora and fauna.

[9] Foissner W., Strüder-Kypke M., van der Staay G.W.M., van der Staay S.-Y., Hackstein J.H.P. (2003). Endemic ciliates (Protozoa, Ciliophora) from tank bromeliads (Bromeliaceae): a combined morphological, molecular, and ecological study. Eur J Protistol.

[10] Gies E.A., Konwar K.M., Beatty J.T., Hallam S.J. (2014). Illuminating microbial dark matter in meromictic Sakinaw Lake. Appl Environ Microbiol.

[11] Goffredi S.K., Kantor A.H., Woodside W.T. (2011). Aquatic microbial habitats within a neotropical rainforest: bromeliads and pH-associated trends in bacterial diversity and composition. Microb Ecol.

[12] Goffredi S.K., Jang G., Woodside W.T., Ussler W. (2011). Bromeliad catchments as habitats for methanogenesis in tropical rainforest canopies. Front Microbiol.

[13] Graham E.A., Andrade J.L. (2004). Drought tolerance associated with vertical stratification of two co-occurring epiphytic bromeliads in a tropical dry forest. Am J Bot.

[14] Greeney H.F. (2001). The insects of plant held waters: a review and bibliography. J Trop Ecol.

[15] Guimarães-Souza B.A., Mendes G.M., Bento L., Marotta H., Santoro A.L., Esteves F.A. (2006). Limnological parameters in the water accumulated in tropical bromeliads. Acra Limnol Bras.

[16] Haroon M.F., Hu S., Shi Y., Imelfort M., Keller J., Hugenholtz P. (2013). Anaerobic oxidation of methane coupled to nitrate reduction in a novel archaeal lineage. Nature.

[17] Hartman W.H., Richardson C.J., Vilgalys R., Bruland G.L. (2008). Environmental and anthropogenic controls over bacterial communities in wetland soils. Proc Natl Acad Sci.

[18] Haubrich C.S., Pires A.P.F., Esteves F.A., Farjalla V.F. (2009). Bottom-up regulation of bacterial growth in tropical phytotelm bromeliads. Hydrobiologia..

[19] Heino J., Virkkala R., Toivonen H. (2009). Climate change and freshwater biodiversity: detected patterns, future trends and adaptations in northern regions. Biol Rev.

[20] Hoekman D. (2010). Turning up the heat: temperature influences the relative importance of top-down and bottom-up effects. Ecology.

[21] Horner-Devine M.C., Carney K.M., Bohannan B.J.M. (2004). An ecological perspective on bacterial biodiveristy. Proc R Soc Lond B.

[22] Hyatt D., Chen G.-L., LoCascio P., Land M., Larimer F., Hauser L. (2010). Prodigal: prokaryotic gene recognition and translation initiation site identification. BMC Bioinformatics.

[23] Jocque M., Field R. (2014). Aquatic invertebrate communities in tank bromeliads: how well do classic ecological patterns apply?. Hydrobiologia.

[24] Kitching R.L. (2001). Food webs in Phytotelmata: “bottom-up” and “top-down” explanations for community structure. Ann Rev Entomol.

[25] Kopylova E., Noé L., Touzet H. (2012). SortMeRNA: fast and accurate filtering of ribosomal RNAs in metatranscriptomic data. Bioinformatics.

[26] Li H., Durbin R. (2009). Fast and accurate short read alignment with Burrows–Wheeler transform. Bioinformatics.

[27] Little T.J., Hebert P.D.N. (1996). Endemism and ecological islands: the ostracods from Jamaican bromeliads. Freshw Biol.

[28] Marino N.A.C., Srivastava D.S., Farjalla V.F. (2013). Aquatic macroinvertebrate community composition in tank-bromeliads is determined by bromeliad species and its constrained characteristics. Insect Conserv Divers.

[29] Markowitz V.M., Chen I.-M.A., Palaniappan K., Chu K., Szeto E., Grechkin Y. (2012). IMG: the integrated microbial genomes database and comparative analysis system. Nucleic Acids Res.

[30] Martinson G.O., Werner F.A., Scherber C., Conrad R., Corre M.D., Flessa H. (2010). Methane emissions from tank bromeliads in neotropical forests. Nat Geosci.

[31] Mason O.U., Hazen T.C., Borglin S., Chain P.S.G., Dubinsky E.A., Fortney J.L. (2012). Metagenome, metatranscriptome and single-cell sequencing reveal microbial response to Deepwater Horizon oil spill. ISME J.

[32] Naeem S. (1988). Predator–prey interactions and community structure: Chironomids, mosquitoes, and copepods in *Heliconia imbricata* (Musaceae). Oecologia.

[33] Ngai J.T., Srivastava D.S. (2006). Predators accelerate nutrient cycling in a bromeliad ecosystem. Science.

[34] Paolotti M.G., Taylor R.A.J., Stinner B.R., Stinner D.H., Benzing D.H. (1991). Diversity of soil fauna in the canopy and forest floor of a Venezuelan cloud forest. J Trop Ecol.

[35] Petrin Z., Laudon H., Malmqvist B. (2007). Does freshwater macroinvertebrate diversity along a pH-gradient reflect adaptation to low pH?. Freshw Biol.

[36] Quast C., Pruesse E., Yilmaz P., Gerken J., Schweer T., Yarza P. (2013). The SILVA ribosomal RNA gene database project: improved data processing and web-based tools. Nucleic Acids Res.

[37] Qi M., Wang P., O'Toole N., Barboza P.S., Ungerfeld E. (2011). Snapshot of the eukaryotic gene expression in muskoxen rumen—a metatranscriptomic approach. PLoS One.

[38] Richardson B.A. (1999). The bromeliad microcosm and the assessment of faunal diversity in a neotropical forest. Biotropica.

[39] Richardson B.A., Rogers C., Richardson M.J. (2000). Nutrients, diversity, and community structure of two phytotelm systems in a lower montane forest, Puerto Rico. Ecol Entomol.

[40] Sabaugh L.T., Dias R.J.P., Branco C.W.C., Rocha C.F.D. (2011). News records of phoresy and hyperphoresy among treefrogs, ostracods, and ciliates in bromeliad of Atlantic forest. Biodivers Conserv.

[41] Shrestha P.M., Kube M., Reinhardt R., Liesack W. (2009). Transcriptional activity of paddy soil bacterial communities. Environ Microbiol.

[42] Srivastava D.S., Trzcinski M.K., Richardson B.A., Gilbert B. (2008). Why are predators more sensitive to habitat size than their prey? Insights from bromeliad insect food webs. Am Nat.

[43] Tews J., Brose U., Grimm V., Tielborger K., Wichmann M.C., Schwager M., Jeltsch F. (2004). Animal species diversity driven by habitat heterogeneity/diversity: the importance of keystone structures. J Biogeogr.

[44] Urich T., Lanzen A., Qi J., Huson D.H., Schleper C., Schuster S.C. (2008). Simultaneous assessment of soil microbial community structure and function through analysis of the meta-transcriptome. PLoS One.

[45] Weygoldt P. (1980). Complex brood care and reproductive behavior in captive poison-arrow frogs, *Dendrobates pumilio*. Behav Ecol Sociobiol.

[46] Winkler U., Zotz G. (2008). Highly efficient uptake of phosphorus in epiphytic bromeliads. Ann Bot.

[47] Zakrzewski M., Goesmann A., Jaenicke S., Jünemann S., Eikmeyer F., Szczepanowski R. (2012). Profiling of the metabolically active community from a production-scale biogas plant by means of high-throughput metatranscriptome sequencing. J Biotechnol.

