# Supplementary figures and images for: Transcriptomics in the tropics: Total RNA-based profiling of Costa Rican bromeliad-associated communities
Source: Comput Struct Biotechnol J. 2014 Dec 13;13:18–23. doi: 10.1016/j.csbj.2014.12.001 (PMC4352299; doi:10.1016/j.csbj.2014.12.001)

Goffredi et al. - Figure 1

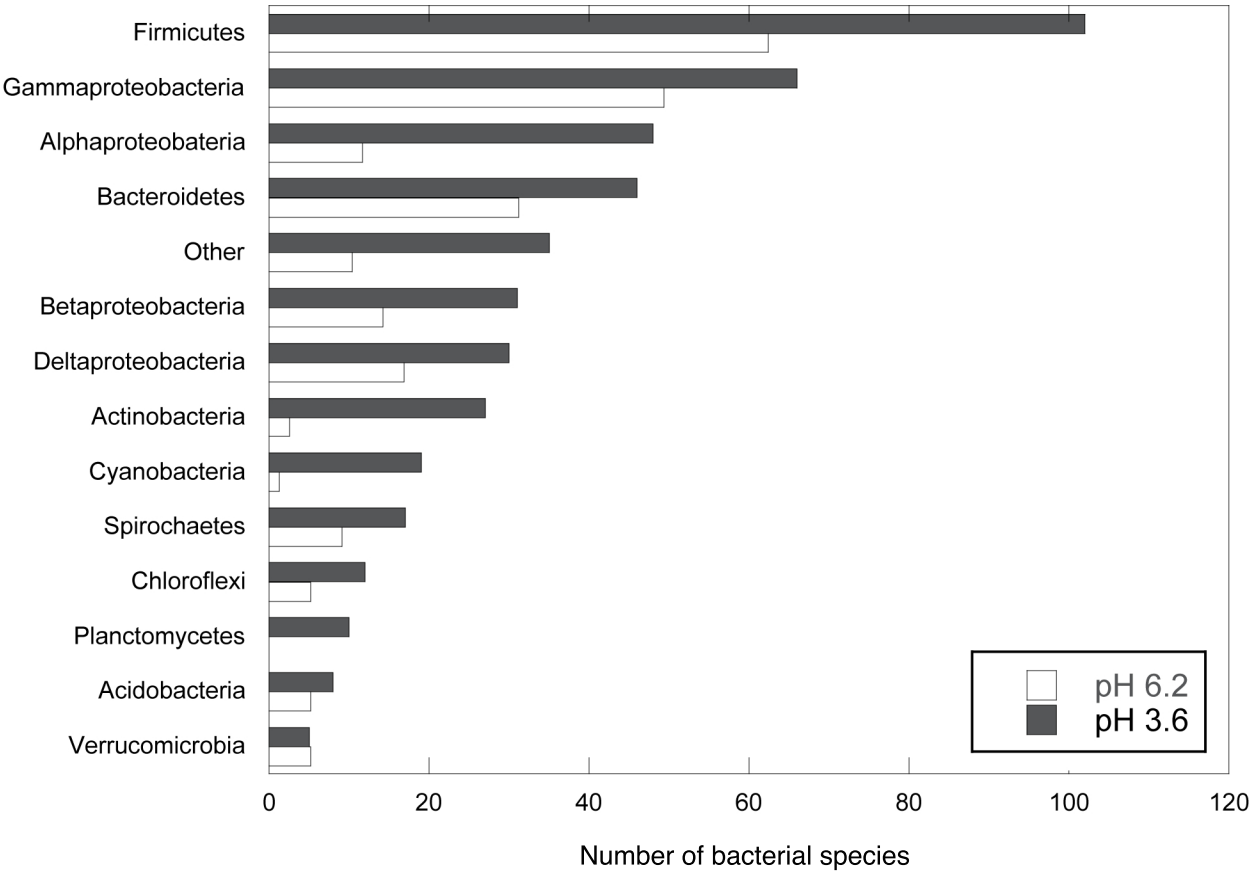

Supplement: Supplemental Fig. 1 — Number of bacterial species represented in metatranscriptomic libraries from two bromeliad tanks. For Wg25, with a pH of 6.2, species number for each phylum was multiplied by 1.3 (from Table 2) to account for a difference in total reads analyzed (16.0 million versus 17.2 million reads, respectively) or contigs recovered (1938 versus 2531), as compared to Wg24 with a pH of 3.6, respectively. [file mmc1.pdf]
